# Computed Tomography Guided Coil Localization and Uniportal Video-Assisted Thoracic Surgery Resection of Small Lung Lesions

**DOI:** 10.7759/cureus.84355

**Published:** 2025-05-18

**Authors:** Yue Li, Shao J Ong, G Anil, Harish Mithiran, Prapul C Rajendran, Chee W Yap, John K Tam, Bernard B Wee

**Affiliations:** 1 Cardiac, Thoracic, and Vascular Surgery, National University Heart Centre, Singapore, SGP; 2 Radiology, National University of Singapore, Singapore, SGP; 3 Radiology, National University Hospital, Singapore, SGP; 4 Thoracic Surgery, Gleneagles Hospital, Singapore, SGP; 5 Surgery, National University of Singapore, Singapore, SGP; 6 Radiology, Ng Teng Fong General Hospital, Singapore, SGP

**Keywords:** coiling, interventional radiology, lung coiling, lung neoplasms, lung nodule, uvats, u-vats, video-assisted thoracoscopic surgery

## Abstract

Purpose

We present our experience with computed tomography (CT) guided coil localization of small pulmonary nodules (<1 cm) followed by Uniportal video-assisted thoracic surgery (UVATS) image-guided resection of these lesions.

Method

We present 61 patients who underwent CT-guided coil localization for 67 lung nodules, followed by image-guided UVATS wedge resection of these nodules from March 2015 to May 2019. Coils were placed first under local anaesthetic by interventional radiology before the patient was transferred to the operating theatre and underwent fluoroscopy-guided wedge resection under general anaesthesia.

Results

All cases had successful surgical resection with negative resection margins. Conclusive histological diagnosis was reached in all cases. Two patients (3%) encountered complications from CT-guided coil localization. One suffered a pneumothorax requiring immediate chest drain insertion, and another required conversion to open thoracotomy due to a dislodged coil.

Conclusions

CT-guided coil localization of pulmonary nodules followed by UVATS wedge resection is a fast and safe procedure that accurately diagnoses and treats small pulmonary nodules.

## Introduction

The advent of computerized tomography (CT) has revolutionized pulmonary imaging and is fast replacing chest radiographs (CXR) for screening and oncological follow-up. CT can detect subtle abnormalities often missed by CXR, such as incidental pulmonary nodules [1, 2]. This has brought about guidelines detailing the management of such nodules [3]. In addition to the patient’s history, CT morphology can suggest a benign or malignant aetiology and guide follow-up management. However, this alone is never confirmatory [4, 5]. Once a nodule reaches a certain size or displays certain characteristics, it is recommended that they are biopsied or resected for histological diagnosis [3].

Bronchoscopy-guided, or CT-guided percutaneous biopsy, is often limited by the size of the lesion and its location [6]. Surgical removal of such suspicious lung nodules by video-assisted thoracic surgery (VATS) has been increasingly employed as a more reliable method of sampling small nodules [7]. Compared to open thoracotomy, VATS offers lower complication rates, less morbidity, and decreased length of stay (LOS) [7]. Current guidelines recommend the removal of nodules more than 8mm in high-risk patients [3]. However, these lesions are difficult to identify visually or by finger palpation due to their small size or non-solid nature [8]. Several techniques have been described to localize these small lesions, such as CT-guided percutaneous injection of compounds (methylene blue, radiotracers, indocyanine green), insertion of hook wires, metallic coils, and sutures. Most of these techniques have variable adoption rates, based on the availability of expertise, resources, and surgical preferences [9-12]. We present our single-institution experience of CT-guided coil localization followed by UVATS resection for small lung lesions in 61 patients from March 2016 to May 2019.

## Materials and methods

Patient selection

Patients were deemed suitable for the coil localization if they fulfilled the following criteria: solitary or multifocal pulmonary lesion(s) more than 5mm in diameter, radiologically suspicious of malignancy (irregular or spiculated margins, solid, subsolid, or ground glass in appearance), and considered impalpable. These lung nodules would have to be accessible via a thoracoscopic approach and be resected with adequate negative margins. Cases were discussed in a multidisciplinary meeting involving thoracic surgeons, interventional radiologists, and respiratory physicians. Patients with biopsy-proven primary lung carcinoma, subpleural surface lesions, and lesions in locations not amenable to wedge resections, such as those close to the bronchus, pulmonary artery, or pulmonary vein, were excluded. Informed consent was obtained from each patient.

Interventional radiology coiling technique

CT-guided coil localization and UVATS resection were performed on the same day. All patients were admitted on the same morning and brought first to interventional radiology for lesional coil localization.

Patients are positioned on the CT scanner either prone or supine, depending on the position of the nodule. The ideal patient position is chosen to ensure the least amount of lung traversed as well as to avoid the fissures. Following a pre-procedural CT thorax to confirm the size and location of the nodule, the patient is cleaned and draped to provide an aseptic procedural field. Local anaesthetic (5-10 ml lidocaine 1%) is administered from the skin to the edge of the pleura. A small incision is made and a 19G access needle (Cook Medical, Bloomington, Indiana) is advanced to the lesion and position confirmed by CT localisation. The tip of the access needle is ideally sited medially and adjacent to the target nodule. Direct placement within the nodule can also be performed, although this potentially increases the diagnostic complexity for the pathologist.

**Figure 1:**
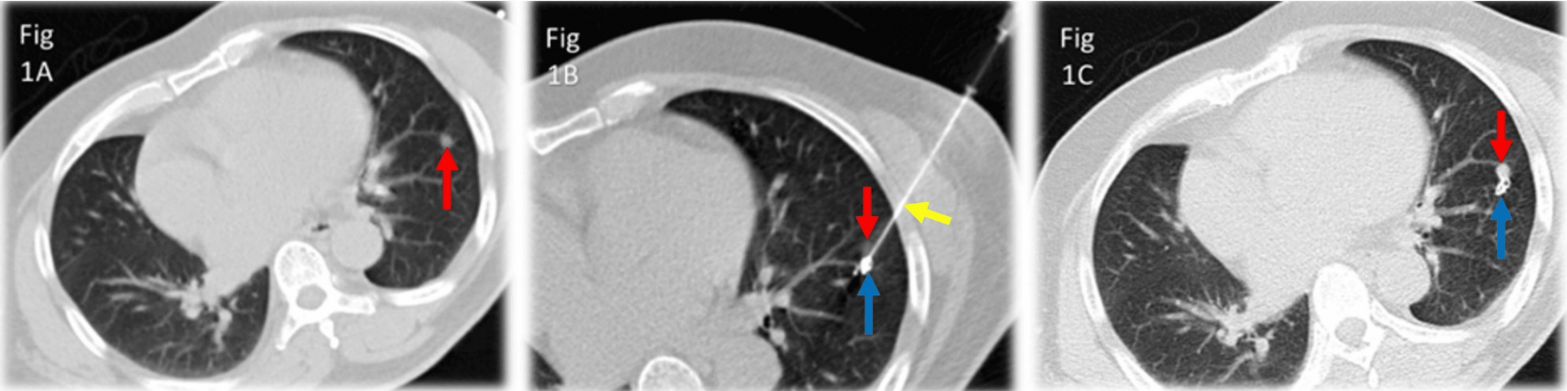
Coil localization of sub-centimeter lung nodule Figure 1A shows the location of a small nodule within the left lower lobe (red arrow). Figure 1B shows the access needle in place (yellow arrow) with the embolization coil (blue arrow) deployed adjacent to the small lung nodule (red arrow). Figure 1C shows the location of the embolization coil (blue arrow) adjacent to the lung nodule (red arrow) after withdrawal of the access needle.

Once the needle is in position, a 5 mm x 50 mm pushable embolization coil (Cook Medical, Bloomington, Indiana) is deployed through the coaxial needle using a straight 0.35-inch wire as a coil pusher. A repeat limited CT is performed to confirm coil location (Figure 1).

The access needle is then withdrawn to the pleural surface, and Methylene blue is injected to mark the entry site of the needle. Only 0.5 ml is injected as excessive amounts tend to flood the lung and alveoli, resulting in excessive coughing as well as pleuritic pain if it spills into the pleural cavity. A CXR is then performed after the localization to confirm coil placement and look for acute complications, and the patient is transferred to the operating theatre for the subsequent lung resection.

**Figure 2:**
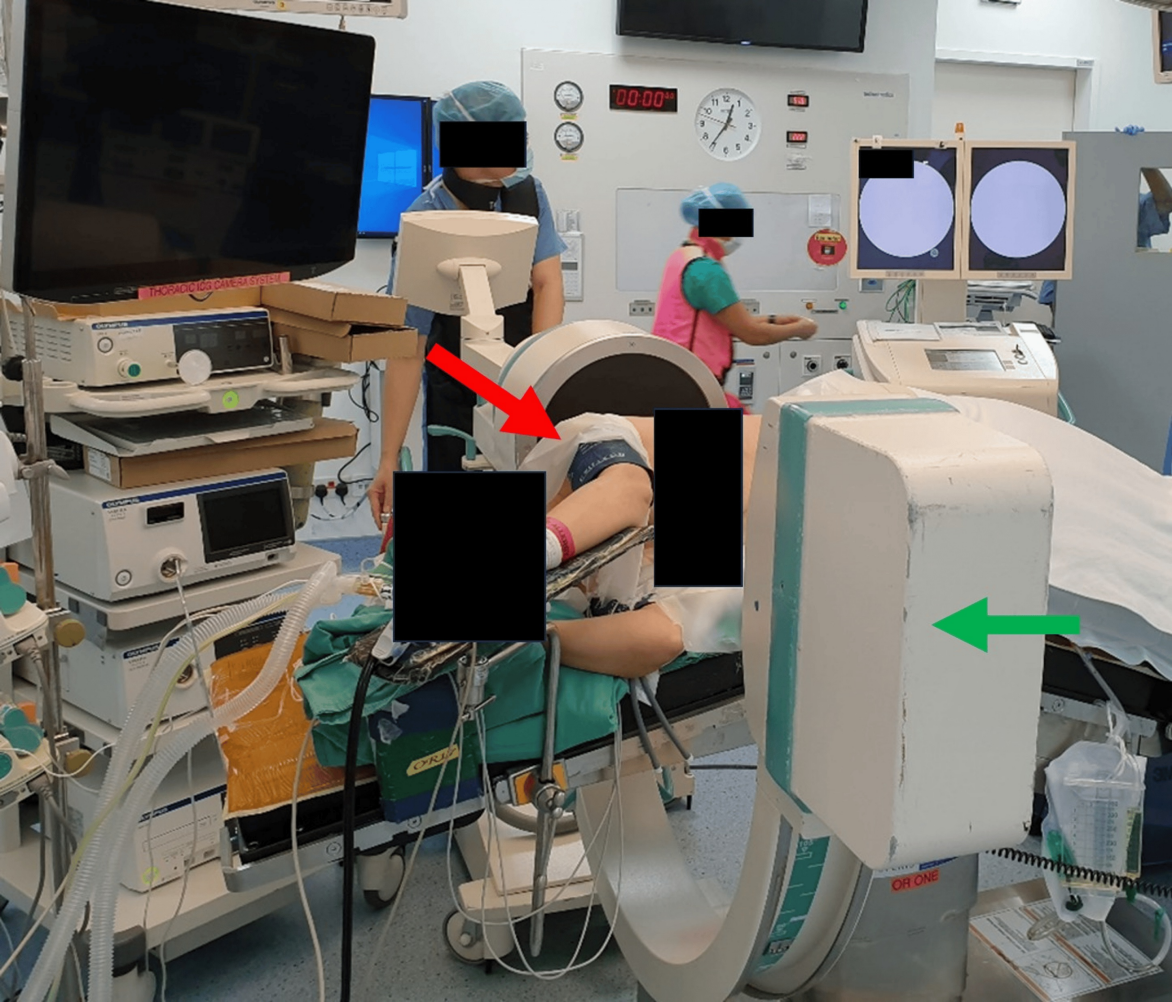
Positioning of patient for Uniportal video-assisted thoracic surgery within the operating theatre with the patient in a lateral decubitus position (red arrow) and the portable image intensifier (green arrow) placement

Once in the operating theatre, the patient is placed under general anaesthesia with double-lumen tube intubation and single-lung ventilation. The patient is positioned in a lateral decubitus position with the lesion side up. Immediately after, the portable image intensifier or C-ring in a hybrid theatre setting is positioned approaching the patient posteriorly (Figure 2).

This position is then locked in and avoids intra-operative collision of the fluoroscopy with the table or patient, risking contamination. The patient and the images intensifier are then cleaned and draped in usual fashion.

**Figure 3:**
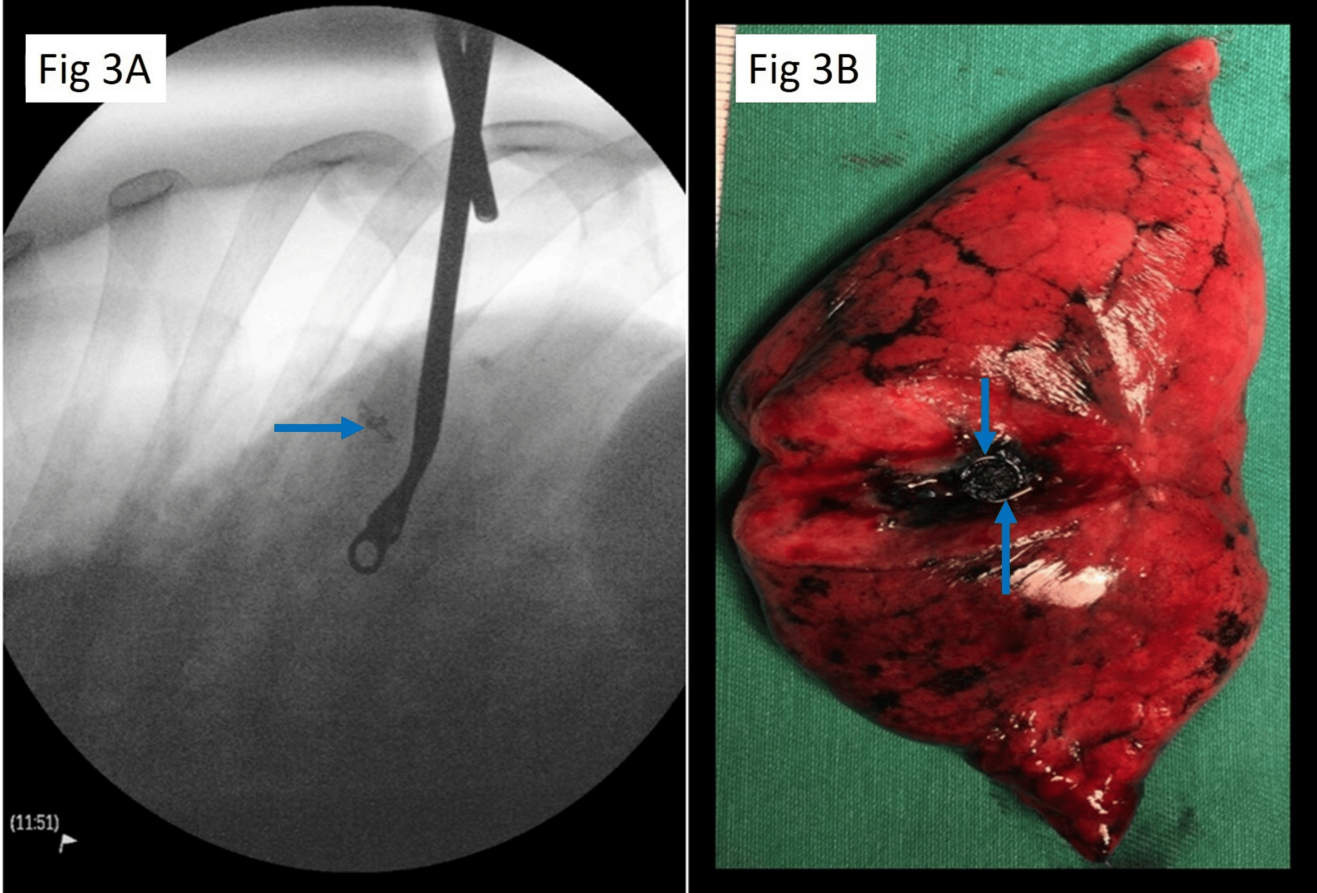
Intraoperative localisation (3A) of the target coiled (blue arrow) nodule and visual conformation (3B) of nodule and coil (blue arrow) within the resected lung segment.

A UVATS incision is made over the mid-axillary line, fourth or fifth intercostal space, 3-4 cm in length. Thoracoscopic examination is performed to assess for pleural metastases and identify the needle entry site via methylene blue. Fluoroscopy is then utilised to localise the coil within the deflated lung. Wedge resection is performed with multiple stapler firings under fluoroscopy guidance deep to the coils. This ensures that the specimen is taken with adequate margins. After delivery of the specimen, a fluoroscopic image and/or direct visualisation is done of the specimen to ensure the coil is removed from the patient (Figure 3). Frozen sections for pathology were performed at the surgeon's discretion.

Postoperatively, a size 20 Fr chest tube is sited, and the patient is extubated to the recovery room where they are monitored for two hours. A chest radiograph would be repeated in the recovery area, confirming the chest tube position and full lung re-expansion before transferring patients back to the ward. Subsequently, the chest tubes were removed the following day if there was no air leak, pleural fluid drainage was not haemorrhagic, and under 300 ml/day. All patients had a repeat CXR immediately after chest drain removal and were either discharged on the same day or the day after.

Study design

We reviewed retrospective data of 61 patients who underwent CT-guided coil localization followed by fluoroscopically guided UVATS resection of pulmonary nodules at our institution in Singapore, from March 2015 to May 2019. The Institutional Domain, Specific Review Board, approved this study with waiver of consent (DSRB reference number 2018/00101).

Demographic data of the patients, medical history, and radiological characteristics of the lesions were collected. Intraoperative details of the procedures were also collated. The postoperative course of the patients was reviewed, including pathological results.

## Results

Demographics and preoperative characteristics

A total of 61 patients with a median age of 62 and 67 lung nodules were included. Six patients had two lesions which were coiled, and the remaining 56 had coiling of a single lesion. Fifteen (24.6%) patients had a history of smoking.

Among the lesions, 40 (59.7%) of the target lesions were solid nodules, 25 (37.3%) were ground-glass opacities (GGOs), and two (3.0%) were cavitary lesions. Mean lesion size was 0.97 ± 0.39 cm and distance to the nearest pleural surface was 0.84 ± 0.84 cm (Table 1).

**Table 1:** Demographics and preoperative characteristics

Characteristics	N (%)
Total patients	61
Total target lesions	67
Gender	
Male	25 (41.0%)
Female	36 (59.0%)
Age	
Less than 60	27 (44.3%)
60-70	28 (45.9%)
Above 70	6 (9.8%)
Smoking history	
Current smoker	6 (9.8%)
Ex-smoker	9 (14.8%)
Never smoker	46 (75.4%)
Lesion characteristics on CT	
Solid nodules	40 (59.7%)
GGO	25 (37.3%)
Cavitatory lesions	2 (3.0%)
Lesion size	0.97±0.39cm
Distance to pleura	0.84±0.84cm

Intraoperative characteristics

The mean waiting time from completion of coiling localization to the start of surgery was 191 ± 150 min, and the mean operative time was 101 ± 56 min. One patient required a chest drain insertion after coiling to relieve a pneumothorax. Of the 61 patients who underwent surgical resection, 20 required further pulmonary resections in the same setting for completion lobectomy, segmentectomy after frozen section was done, or additional wedge resections. One patient required a conversion to open thoracotomy, as the coil was dislodged and could not be identified via fluoroscopy.

Postoperative outcomes

Formal histology was obtained for all patients (Table 2). All resection margins were free of lesional tissue. Non-malignant lesions were present in 17 (25.4%) of the resected samples, metastasis in 29 (43.3%) lesions, primary invasive lung cancer was present in 12 (17.9%) lesions, lung adenocarcinoma-in-situ in four (6.0%) lesions, and minimally invasive adenocarcinoma in five (7.5%) lesions. 

**Table 2:** Postoperative outcomes from coil localization uniportal video-assisted thoracic surgery (UVATS) resection of small lung lesions

Outcomes	N (%)
Post-coiling chest tube insertion	1 (1.5%)
Coil dropped out after lung isolation	1 (1.5%)
Additional lung resection performed	20 (29.9%)
Median length of postoperative stay	3 days
Frozen section performed	18 (26.9%)
Primary lung carcinoma	7 (10.4%)
Neoplasm with uncertain origin	3 (4.5%)
Metastatic malignancies	2 (3.0%)
Benign lesions	6 (9.0%)
Final histology	
Benign lesion	17 (25.4%)
Metastatic lesion	29 (43.3%)
Primary invasive lung cancer	12 (17.9%)
Lung adenocarcinoma-in-situ	4 (6.0%)
Minimally invasive adenocarcinoma	5 (7.5%)
Margin status	
Positive	0
Negative	67 (100%)
Postoperative complications	7 (11.5%)
Pneumonia	3 (4.9%)
Pigtail insertion	3 (4.9%)
Prolonged air leak	1 (1.6%)

The median length of stay after surgery was three days. Complications occurred in seven patients. Three developed postoperative pneumonia requiring antibiotics, three developed pneumothorax post chest drain removal, requiring placement of a chest tube. One patient had a prolonged air leak for more than seven days and was discharged with a Heimlich valve.

## Discussion

One of the earliest methods of localization used a hookwire, as described by Gossot et al., where a breast localization wire was used to localize small peripheral pulmonary nodules [11]. These hookwires were, however, prone to dislodgement during patient transfer or lung isolation because part of the wire was external to the chest. Dislodgement occurred in up to 7.5% of cases [13, 14]. Another localization technique is an injection of various types of contrast media or pigmented material or both, using a percutaneous or bronchoscopy approach. However, nearly a fifth of cases fail due to diffusion of the dye in the pleural space or lack of visualization of the dye intraoperatively [15].

Coil localization with CT guidance was first introduced by Powell et al. in 2004 [10]. We adapted this technique as we found it to be advantageous over other techniques. The coil is readily available, radiopaque, and easily identifiable on fluoroscopy, non-dissolvable, and less prone to migration after deployment and on lung isolation. Combining this with UVATS allows for lung resections via a single small incision without rib spreading. It has favourable outcomes with regard to postoperative analgesic usage, length of hospitalization, thoracostomy drainage duration, and morbidity [16, 17]. Our technique also does not require the use of a hybrid theatre. A portable image intensifier fluoroscopy is sufficient for the detection of the coils and ensuring that they are removed in their entirety with the specimen.

Image-guided coil localization ensures accurate identification of lesions for resection. A randomized control trial by Finley et al. compared non-localized VATS resection and coil-localized VATS resection in 56 patients. The result showed that coil insertion before VATS wedge resection resulted in an improved diagnostic rate by 1.93-fold [18]. In our study, we achieved a 100% resection rate with histology diagnosis and clear margins. This may have been contributed by the level operators' experience in addition to the technique. The lung coilings were only performed by consultant interventional radiologists who routinely perform lung biopsies. The surgical resections were only performed by cardiothoracic surgical consultants who specialise in minimally invasive thoracic surgery. Performing procedures following this system avoids repeat operations for positive margins or missed lesions. We believe this is an improvement over non-localized VATS resection in small lung lesions.

Coiling has been proven safe in several retrospective studies with a low complication rate of 3-17% during the procedure [19-24]. In our series, the complication rate following coil insertion was 3% (n=2), with one patient having a symptomatic pneumothorax requiring a chest drain insertion and another requiring conversion to a lateral thoracotomy as the coil was not localized intraoperatively and not visible on fluoroscopy. The coil was superficially placed near the visceral pleura, and we believe the coil was dislodged during lung isolation and removed by initial suctioning of the pleural cavity. After this incident, we modified our technique so that all coils were placed deep to the lesion to avoid dislodgement and achieve adequate resection margins.

In our centre, the mean time between the completion of the coil insertion (time of last CT scan) and the start of operation (time of patient entering the OR) was 191 ±150 min. A recent retrospective analysis by Donahoe et al. showed their waiting time was 136.6±89.0 min [19]. The main reason for the prolonged waiting time in our centre was that the coils were percutaneously inserted in the morning at the radiology department, but were listed for surgery as the second or third case of the day, which was usually in the afternoon. Once the micro coils were inserted, patients would be transferred back to the ward or the operating theatre. This increased the waiting time between procedures but did not have any negative effect on the patients. It also allowed better utilization of scarce operating theatre and interventional suite resources. To have a more seamless approach, some centres perform localization of these lung nodules, followed by image-guided VATS resection in a hybrid operating room at the same setting [25].

Studies have reported that the coil-guided VATS resection mean operative time ranged from 52 to 89 min in past studies [10,19,20]. In our study, it was 101±56 min, which was longer than in other reviews. This extended time was due to a third of the patients (20 out of 61 patients) undergoing further pulmonary resections in the same setting. Excluding these combined procedures, the mean operative time for a single-coil guided UVATS wedge resection was 73 ± 30 min, which was comparable to other reported past studies [10, 19, 20].

## Conclusions

CT-guided coil localization is an advantageous procedure that increases the success of completely resecting the small pulmonary lesions via UVATS. Performing the lung nodule coiling in interventional radiology first, followed by the same day lung resection, allowed for maximal usage of scarce interventional rabdology suite resources and surgical operating theatre time slots. The waiting time between the lung coiling procedure and the UVATS procedure did not result in any complications.
